# Geographic and socioeconomic disparity in cardiovascular risk factors in Indonesia: analysis of the Basic Health Research 2018

**DOI:** 10.1186/s12889-020-09099-1

**Published:** 2020-06-26

**Authors:** Wiku Adisasmito, Vilda Amir, Anila Atin, Amila Megraini, Dian Kusuma

**Affiliations:** 1 Faculty of Public Health, University of Indonesia, Depok, Indonesia; 2 Indonesia One Health University Network (INDOHUN), University of Indonesia, Depok, Indonesia; 3 Centre for Health Economics and Policy Innovation, Imperial College Business School, South Kensington Campus, London, SW7 2AZ UK

**Keywords:** Cardiovascular risk factors, Geographic, Socioeconomic, Disparity, Indonesia

## Abstract

**Background:**

Cardiovascular diseases (CVDs) accounted for over 17 million deaths and 353 million disability-adjusted life years lost in 2016. The risk factors are also high and increasing with high blood pressure, smoking, and high body mass index contributed to up to 212 million disability-adjusted life years in 2016. To help reduce the burden, it is crucial to understand the geographic and socioeconomic disparities in CVD risk factors.

**Methods:**

Employing both geospatial and quantitative analyses, we analyzed the disparities in the prevalence of smoking, physical inactivity, obesity, hypertension, and diabetes in Indonesia. CVD data was from Riskesdas 2018, and socioeconomic data was from the World Bank.

**Results:**

Our findings show a very high prevalence of CVD risk factors with the prevalence of smoking, physical activity, obesity, hypertension ranged from 28 to 33%. Results also show the geographic disparity in CVD risk factors in all five Indonesian regions. Moreover, results show socioeconomic disparity with the prevalence of obesity, hypertension, and diabetes are higher among urban and the richest and most educated districts while that physical inactivity and smoking is higher among rural and the least educated districts.

**Conclusion:**

The CVD burden is high and increasing in particularly among urban areas and districts with higher income and education levels. While the government needs to continue tackling the persistent burden from maternal mortality and infectious diseases, they need to put more effort into the prevention and control of CVDs and their risk factors.

## Background

Cardiovascular diseases (CVDs) accounted for over 17 million deaths and 353 million disability-adjusted life years (DALYs) lost with ischemic heart disease and stroke as the first and second leading causes of DALYs in 2016 [1, 2]. The burden of CVD risk factors is also high and increasing. High blood pressure, smoking, and high body mass index (BMI) contributed to over 212 million, 155 million, and 135 million DALYs in 2016, respectively. Smoking and high blood pressure were the leading risks among men, while high blood pressure and high BMI were the leading risks among women in 2016 [3].

Like many low and middle-income countries (LMICs), the burden of CVDs and risk factors is high and increasing in Indonesia, a lower-middle-income country [4]. The Global Burden of Disease study shows that stroke and ischemic heart disease are the top causes of deaths and disability in 2017 [5]. The Basic Health Research (Riskesdas), a nationally representative health survey, showed that the prevalence of diagnosed stroke among people aged 15 years and above increased by 56% (from 0.7 to 1.1%) during 2013–2018. The prevalence of hypertension among people aged 18 years and above also increased by 32% (from 26 to 34%), and that of obesity increased by 47% (from 15 to 22%) during 2013–2018 [6].

To achieve the Sustainable Development Goals on reducing premature deaths from non-communicable diseases, reduction in geographic and socioeconomic disparity in the burden of CVDs and risk factors is crucial [7, 8]. Evidence from high-income countries shows while the socioeconomic gap in life expectancy is narrowing, the disparity in CVD mortality and risk factors such as smoking, obesity, and hypertension are widening [9, 10]. However, current studies on geographic and socioeconomic inequality in CVD risk factors are limited in three ways. First, many studies are from high-income countries, including the United States, the United Kingdom, and South Korea [10–18]. Second, the few studies from LMICs were socioeconomic inequalities in BMI and obesity focusing among women [19–21], and lacking evidence on geographic disparity [22–24]. In this paper, we aim to address this evidence gap by examining the geographic and socioeconomic disparity in CVD risk factors in Indonesia.

## Methods

### Study design and sampling

This is a cross-sectional study on the geographic and socioeconomic disparity in CVD risk factors among districts in Indonesia. Data for CVD risk factors were from the district-level aggregate data of Riskesdas 2018, a nationally representative health survey conducted by the Ministry of Health. The target sample was 300,000 households from 30,000 census blocks (CBs) from the National Socioeconomic Survey (Susenas) with two-stage sampling. In the first stage, out of a total of 720.000 CBs from the 2010 population census, 180.000 CBs (25%) were selected using probability proportional to size. In each urban and rural stratum, 30,000 CBs were selected using probability proportional to size. In the second stage, 10 households were systematically selected using implicit stratification of education level of household head, to maintain variations among households. In each household, each member was interviewed, but only members meeting the criteria were selected for examination, including those 15+ years old for blood glucose and lipid profile. The interview response rate was 95% of target households at the national level, ranging from 85% in Papua to 99% in Bangka Belitung province. There were 818,507 and 713,783 individuals aged 10+ and 15+ years old, respectively [25].

Socioeconomic data were from the World Bank. In addition to the geospatial analysis, we conducted quantitative analyses on geographic disparity, including region and urbanicity. The National Planning Agency (Bappenas) divides the 34 provinces into seven regions, including Sumatera, Java, Kalimantan, Sulawesi, Nusa Tenggara, Maluku, and Papua. However, we combined the last three as one region (Papua) because they have fewer districts and similarly the least developed in the country. Moreover, we conducted quantitative analyses on socioeconomic disparity, including income and education indicators by urban/rural. We defined cities as urban and regents as rural; we used district-level poverty rate for income with the lowest rate as quintile 5; and we used the net enrollment ratio of senior secondary for education with the highest ratio as quintile 5.

### Dependent variables

There are five main CVD risk factors as the dependent variables, including the prevalence of smoking, physical inactivity, obesity, hypertension, and diabetes mellitus. Smoking was defined as current smoking status of respondents aged 10 years and above. Physical inactivity was defined as lack of rigorous or moderate activity in the last week per the World Health Organization’s Global Physical Activity Questionnaire (GPAQ) among respondents aged 10 years and above. Rigorous activity is an activity that is carried out continuously for at least 10 min for at least 3 days last week with a total activity duration of at least 1500 metabolic equivalent of task (MET) minute. MET minute of rigorous activity is the duration (minutes) of activities in a week multiplied by eight calories. Moderate activity including activity (e.g., sweeping, mopping, etc.) of at least 5 days with a total duration of 150 min last week. Obesity was measured by central obesity among aged 15 years and above that is abdominal circumference of more than 80 cm in women (excluding pregnant women) and of more than 90 cm in men. Hypertension was among aged 15 years and above with systolic blood pressure of at least 140 mmHg or diastolic of 90 mmHg. Diabetes mellitus was measured among respondents aged 15 years and above who have been diagnosed by a doctor.

### Data analysis

We conducted geospatial analyses by dividing the CVD risk prevalence among 34 provinces and 514 districts into five quintiles in ArcMap 10. Moreover, we conducted bivariate Ordinary Least Square (OLS) regressions in STATA 15 to show associations between geographic (i.e. urban/rural and region) and socioeconomic (i.e. income and education) disparity in each CVD risk factor (smoking, physical inactivity, obesity, hypertension, and diabetes mellitus). We also calculated the absolute and relative differences between geographic and socioeconomic variation. For the region, absolute and relative differences were between Java (most developed) and Papua (least developed). For income and education, absolute and relative differences were between quintile 5 (wealthiest and most educated) and quintile 1 (poorest and least educated).

While age is an important CVD risk factor, there was only district-level age data for 2010 available from the World Bank (based on population census). We used the proportion of population aged 65+ years for each district (in quintile) in 2010, but there were fewer districts. The full regression results without (*n* = 514 districts) and with (*n* = 497 districts) controlling for age are similar, as shown in Additional file 1, respectively. Thus, we presented the former as our main results. All statistical significance was at the 5% level.

## Results

Results will be in two parts: the provincial and district levels. Evidence on the disparity in CVD risk factors at the provincial level is relevant for national development planning, but the number of observations (e.g., 34 provinces) is limited for quantitative analysis. The disparity at the district level is crucial for at least two reasons. First, the decentralization policy in Indonesia that started in 2001 transferred much authority for local development planning (including the health sector) to the mayor of districts (including cities). After 2001, the district health office is accountable to the mayor (and to the Ministry of Home Affairs) instead of previously to the Ministry of Health. Also, the number of observations (e.g., 514 districts) is sufficient for further quantitative analysis.

### Provincial level

**Fig. 1:**
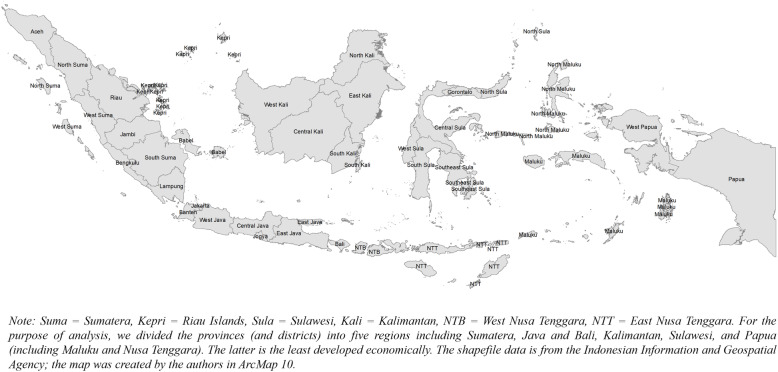
Map of Indonesia by province

**Fig. 2:**
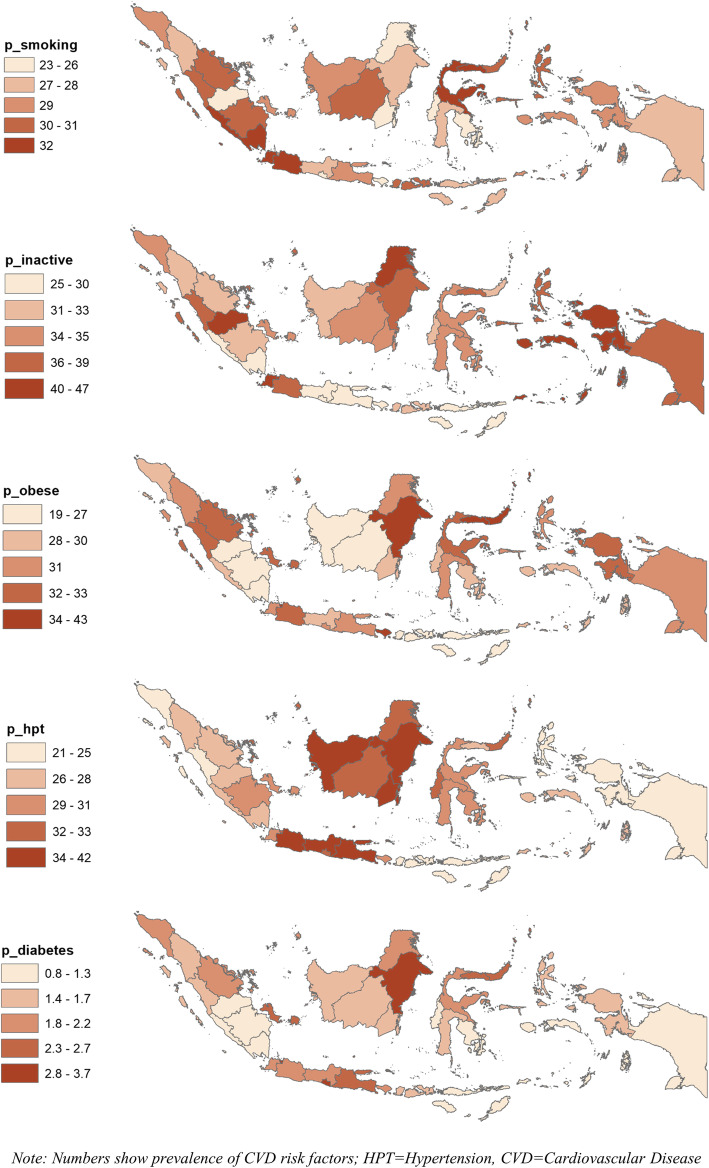
Disparity of CVD risk factors by province in Indonesia, 2018

Figure 1 shows the map of Indonesia, with 34 provinces distributed into five regions of Sumatera, Java (including Bali), Kalimantan, Sulawesi, and Papua (including Maluku and Nusa Tenggara). In general, provinces in the western regions (e.g., Java and Sumatera) are more developed than those in the eastern areas (e.g., Papua and Nusa Tenggara). In terms of geographic disparity, Fig. 2 shows the distribution of CVD risk factor prevalence quintiles. The prevalence of smoking ranged from 23 to 32%; that of inactivity ranged from 25 to 47%; that of obesity ranged from 19 to 43%; that of hypertension ranged from 21 to 42%; that of diabetes ranged from 0.8 to 3.7%. For smoking, the prevalence was highest (quintile 5), particularly in provinces in the southern part of Sumatera (e.g., Lampung), the western part of Java (e.g., Banten), and northern part Sulawesi (e.g., Gorontalo) regions. For obesity, the prevalence was highest in the Kalimantan (e.g., East Kalimantan) and Sulawesi (e.g., North Sulawesi) regions. For hypertension, the prevalence was the highest in most provinces in the Kalimantan and Java regions.

In terms of socioeconomic disparity, Table 1 shows the prevalence of CVD risk factors by the provincial income level. The provinces in the top box (e.g., Bali) are more affluent, and those in the bottom box (e.g., West Papua) are poorer. The shaded prevalence shows higher than the national average for each risk factor. Results show more shaded prevalence among the wealthiest provinces for all risk factors but smoking. Five of the 10 wealthiest provinces have shaded smoking prevalence, while seven of the 10 poorest provinces do. Six of the 10 wealthiest provinces have the shaded hypertension prevalence, while none of the 10 poorest provinces do.

**Table 1:**
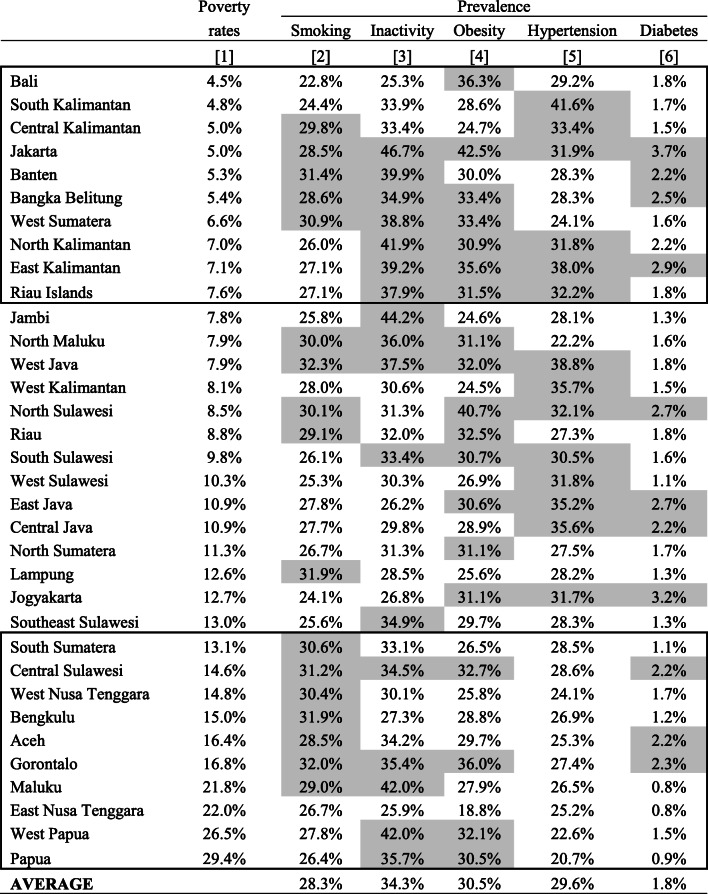
Prevalence of CVD risk factors by province in Indonesia, 2018

Note: Ordered by the average poverty rates (column 1), the provinces in the top box are richest and those in the bottom box are poorest. Shaded values show higher than the national average for each risk factor

### District level

Table 2 shows the characteristics of districts and the prevalence of CVD risk factors. In terms of district characteristics (panel a), the regions of Sumatera and Java have a lot more districts with 154 (30%) and 128 (25%) districts out of a total of 514, respectively. There are 97 urban cities (19%) and 417 rural regents (81%). By income, 79% of urban areas are in the fourth or fifth quintiles (richer) while almost half (48%) of rural areas are in the first and second quintiles (poorer). By education, 71% of the urban areas are in the fourth or fifth quintiles (most educated), while almost half (47%) of rural areas are in the first and second quintiles (least educated). In terms of CVD risk factors (panel b), the prevalence of smoking, physical activity, obesity, hypertension, and diabetes is 28, 33, 30, 30, and 2%, respectively. There is a significant disparity between urban and rural, except for the prevalence of hypertension. While smoking prevalence is lower in urban areas by 6.3% (i.e., 1.7% divided by 27% urban smoking prevalence), the prevalence of physical inactivity, obesity, and diabetes is higher in urban areas by 20.8, 22.4, and 40%, compared to rural areas.

**Table 2:** Characteristics of districts and CVD risk factors

	All		Urban		Rural		Difference
	n	%	n	%	n	%	%
	[1]	[2]	[3]	[4]	[5]	[6]	[7]= [4–6]
(a) Characteristics (#)							
Sample size district	514	100%	97	100%	417	100%	0%
Region							
Papua	95	18%	9	9%	86	21%	−11%
Java	128	25%	35	36%	93	22%	14%
Sumatera	154	30%	33	34%	121	29%	5%
Kalimantan	56	11%	9	9%	47	11%	−2%
Sulawesi	81	16%	11	11%	70	17%	−5%
	514		97		417		
Income/poverty							
Q1 poor	102	20%	3	3%	99	24%	−21%
Q2	103	20%	5	5%	98	24%	−18%
Q3	103	20%	13	13%	90	22%	−8%
Q4	103	20%	22	23%	81	19%	3%
Q5 rich	103	20%	54	56%	49	12%	44%
	514		97		417		
Education							
Q1 least	103	20%	0	0%	103	25%	−25%
Q2	103	20%	11	11%	92	22%	−11%
Q3	103	20%	17	18%	86	21%	−3%
Q4	103	20%	29	30%	74	18%	12%
Q5 most	102	20%	40	41%	62	15%	26%
	514		97		417		
(b) CVD risk factors (%)							
Smoking	n/a	28%	n/a	27%	n/a	29%	−1.7%*
Physical inactivity	n/a	33%	n/a	40%	n/a	32%	8.3%*
Obesity	n/a	30%	n/a	37%	n/a	28%	8.3%*
Hypertension	n/a	30%	n/a	30%	n/a	30%	0.2%
Diabetes mellitus	n/a	2%	n/a	3%	n/a	2%	1.2%*

*CVD* Cardiovascular Diseases, *Q* Quintile, *n* number, *%* proportion of column total, *Urban* City, *Rural* District/Regent. Data on district characteristics are from the World Bank and data on CVD risk factors are from Basic Health Survey 2018. Bold numbers with asterisk (*) show statistically significance at 5% level – full regression results are provided in Additional file 1 panel (a)

**Fig. 3:**
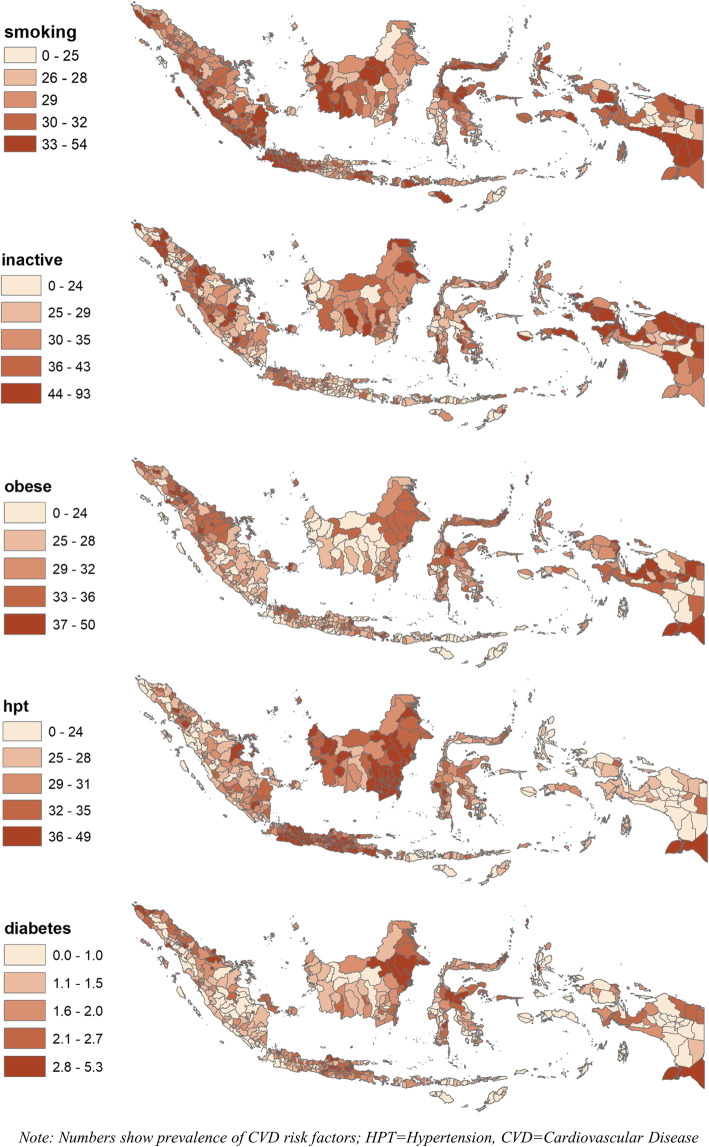
Disparity of CVD risk factors by district in Indonesia, 2018

In terms of geographic disparity, Fig. 3 shows the distribution of district-level CVD risk factor prevalence by quintile. Results show more granularity in the disparity by district, compared to that by province. Many districts in the northern part of Sumatera and several parts of Papua have the highest prevalence of all risk factors, particularly smoking, obesity, and diabetes.

In terms of socioeconomic disparity, Tables 3 and 4 provide the 10 districts with the lowest and highest prevalence. The prevalence of smoking ranged from 5.3% in Yahukimo district (Papua province) to 53.5% in Asmat district (Papua); that of inactivity ranged from 9.4% in Yahukimo district (Papua) to 93.1% in Yalimo district (Papua); that of obesity ranged from 6% in Nias district (North Sumatera) to 49.7% in Karo district (North Sumatera); that of hypertension ranged from 9.6% in Nduga district (Papua) to 49% in Hulu Sungai Tengah district (South Kalimantan); and that of diabetes ranged from 0% in all bottom 10 districts (mostly in Papua provinces) to 5.3% in Madiun city (East Java). By urbanicity, all the bottom districts are rural (except for one), but many of the top districts, particularly for obesity and diabetes, are urban. By income, the average poverty rates among the bottom 10 districts with the highest prevalence of obesity, hypertension, and diabetes are up to 35% while that among the top 10 districts are up to 8%.

**Table 3:** Bottom ten districts with lowest prevalence of CVD risk factor in Indonesia

	Prevalence	Province	Region	Urban	Poverty	Education	Pop (000)
(a) Smoking							
Kab. Yahukimo	5.3%	Papua	Papua	Rural	39%	12%	181
Kab. Nias Selatan	8.0%	North Sumatera	Sumatera	Rural	17%	65%	308
Kab. Nias	12.1%	North Sumatera	Sumatera	Rural	16%	62%	136
Kab. Sarolangun Bangko	13.4%	Jambi	Sumatera	Rural	9%	59%	278
Kab. Nias Barat	15.3%	North Sumatera	Sumatera	Rural	27%	80%	85
Kab. Puncak	15.6%	Papua	Papua	Rural	38%	9%	103
Kab. Paniayi	16.0%	Papua	Papua	Rural	37%	25%	164
Kab. Mambramo Tengah	16.1%	Papua	Papua	Rural	37%	54%	46
Kota Gunungsitoli	16.4%	North Sumatera	Sumatera	Urban	18%	76%	136
Kab. Buton	16.4%	Southeast Sulawesi	Sulawesi	Rural	14%	69%	98
**AVERAGE**					**25%**	**51%**	**154**
(b) Inactivity							
Kab. Yahukimo	9.4%	Papua	Papua	Rural	39%	12%	181
Sabu Raijua	10.7%	East Nusa Tenggara	Papua	Rural	31%	69%	86
Kab. Diyai	10.9%	Papua	Papua	Rural	43%	51%	69
Kab. Bangli	12.9%	Bali	Java	Rural	5%	72%	222
Kab. Kupang	13.0%	East Nusa Tenggara	Papua	Rural	23%	58%	347
Kab. Kulon Progo	13.1%	Yogjakarta	Jawa	Rural	18%	81%	412
Kab. Aceh Jaya	13.3%	Aceh	Java	Rural	14%	74%	86
Kab. Kepahiang	13.3%	Bengkulu	Sumatera	Rural	14%	71%	132
Kab. Pidie	13.7%	Aceh	Sumatera	Rural	20%	74%	418
Kab Bener Meriah	14.3%	Aceh	Sumatera	Rural	20%	67%	137
**AVERAGE**					**23%**	**63%**	**209**
(c) Obesity							
Kab. Nias	6.0%	North Sumatera	Sumatera	Rural	16%	62%	136
Sumba Barat Daya	10.3%	East Nusa Tenggara	Papua	Rural	29%	42%	319
Kab. Manggarai Timur	10.5%	East Nusa Tenggara	Papua	Rural	27%	43%	272
Kab. Nias Barat	10.6%	North Sumatera	Sumatera	Rural	27%	80%	85
Sumba Tengah	11.4%	East Nusa Tenggara	Papua	Rural	35%	44%	68
Kab. Sumba Barat	12.0%	East Nusa Tenggara	Papua	Rural	29%	55%	122
Kab. Nias Selatan	12.4%	North Sumatera	Sumatera	Rural	17%	65%	308
Sabu Raijua	12.9%	East Nusa Tenggara	Papua	Rural	31%	69%	86
Kab. Timor Tengah Selatan	13.2%	East Nusa Tenggara	Papua	Rural	28%	52%	459
Kab. Belu	14.6%	East Nusa Tenggara	Papua	Rural	16%	54%	206
**AVERAGE**					**25%**	**57%**	**206**
(d) Hypertension							
Kab. Nduga	9.6%	Papua	Papua	Rural	38%	9%	94
Kab. Puncak Jaya	10.0%	Papua	Papua	Rural	36%	21%	115
Kab. Tolikara	11.0%	Papua	Papua	Rural	33%	34%	131
Kab. Mambramo Tengah	12.1%	Papua	Papua	Rural	37%	54%	46
Kab. Asmat	12.2%	Papua	Papua	Rural	27%	21%	88
Kab. Sorong Selatan	13.1%	West Papua	Papua	Rural	19%	56%	43
Kab. Lanny Jaya	13.2%	Papua	Papua	Rural	40%	46%	172
Kab. Yahukimo	13.4%	Papua	Papua	Rural	39%	12%	181
Kab. Teluk Wondama	13.4%	West Papua	Papua	Rural	33%	39%	30
Kab. Halmahera Tengah	14.2%	North Maluku	Papua	Rural	14%	63%	50
**AVERAGE**					**32%**	**36%**	**95**
(e) Diabetes							
Kab. Nduga	0.0%	Papua	Papua	Rural	38%	9%	94
Kab. Puncak Jaya	0.0%	Papua	Papua	Rural	36%	21%	115
Kab. Yahukimo	0.0%	Papua	Papua	Rural	39%	12%	181
Kab. Teluk Wondama	0.0%	West Papua	Papua	Rural	33%	39%	30
Kab. Mambramo Raya	0.0%	Papua	Papua	Rural	30%	51%	21
Kab. Jayawijaya	0.0%	Papua	Papua	Rural	39%	67%	206
Kab. Dogiyai	0.0%	Papua	Papua	Rural	30%	39%	92
Kab. Intan Jaya	0.0%	Papua	Papua	Rural	43%	9%	46
Kab. Diyai	0.0%	Papua	Papua	Rural	43%	51%	69
Kab. Buton Selatan	0.0%	Southeast Sulawesi	Sulawesi	Rural	15%	44%	77
**AVERAGE**					**35%**	**34%**	**93**

*CVD* Cardiovascular Disease, *Urban* City, *Rural* District/Regent, *Pop* Population. The districts are ordered by risk factor prevalence

**Table 4:** Top ten districts with highest prevalence of CVD risk factor in Indonesia, 2018

	Prevalence	Province	Region	Urban	Poverty	Education	Pop (000)
(a) Smoking							
Kab. Asmat	53.5%	Papua	Papua	Rural	27%	21%	88
Kab. Mappi	42.7%	Papua	Papua	Rural	26%	16%	92
Kab. Boven Digul	41.5%	Papua	Papua	Rural	20%	35%	63
Kab. OKU Selatan	37.9%	South Sumatera	Sumatera	Rural	11%	61%	344
Kab. Garut	37.6%	West Java	Java	Rural	9%	51%	2547
Kab. Bolaang Mongo Utara	37.3%	North Sulawesi	Sulawesi	Rural	9%	75%	76
Kab. Empat lawang	36.9%	South Sumatera	Sumatera	Rural	12%	62%	238
Kab. Pandeglang	36.9%	Banten	Java	Rural	10%	50%	1194
Kab. Sumedang	36.8%	West Java	Java	Rural	10%	43%	1137
Kab. Bolaang Mongondow	36.8%	North Sulawesi	Sulawesi	Rural	8%	51%	233
**AVERAGE**					**14%**	**46%**	**601**
(b) Inactivity							
Kab. Yalimo	93.1%	Papua	Papua	Rural	35%	28%	59
Kab. Sarolangun Bangko	86.3%	Jambi	Sumatera	Rural	9%	59%	278
Kab. Supiori	64.5%	Papua	Papua	Rural	39%	57%	18
Kab. Raja Ampat	63.9%	West Papua	Papua	Rural	18%	45%	46
Kota Sungai Penuh	62.4%	Jambi	Sumatera	Urban	3%	78%	87
Malaka	62.1%	East Nusa Tenggara	Papua	Rural	16%	58%	180
Kota Ternate	61.7%	North Maluku	Papua	Urban	3%	63%	213
Kab. Pegunungan Bintang	61.6%	Papua	Papua	Rural	31%	21%	72
Kota Bukittinggi	60.3%	West Sumatera	Sumatera	Urban	5%	78%	122
Kab. Maybrat	59.5%	West Papua	Papua	Rural	33%	69%	37
**AVERAGE**					**19%**	**56%**	**111**
(c) Obesity							
Kab. Karo	49.7%	North Sumatera	Sumatera	Rural	9%	74%	389
Kab. Minahasa	48.7%	North Sulawesi	Sulawesi	Rural	7%	65%	329
Kota Pematang Siantar	48.3%	North Sumatera	Sumatera	Urban	9%	77%	247
Kab. Minahasa Selatan	47.8%	North Sulawesi	Sulawesi	Rural	9%	62%	205
Kota Jakarta Pusat	46.9%	Jakarta	Java	Urban	4%	55%	914
Kota Manado	45.6%	North Sulawesi	Sulawesi	Urban	5%	66%	425
Kota Tomohon	45.1%	North Sulawesi	Sulawesi	Urban	6%	71%	100
Kota Padang Panjang	44.5%	West Sumatera	Sumatera	Urban	6%	74%	51
Kota Mojokerto	44.2%	East Java	Java	Urban	6%	80%	126
Kota Gorontalo	44.0%	Gorontalo	Sulawesi	Urban	6%	56%	202
**AVERAGE**					**7%**	**68%**	**299**
(d) Hypertension							
Kab. Hulu Sungai Tengah	49.2%	South Kalimantan	Kalimantan	Rural	6%	66%	260
Kab. Tabalong	48.4%	South Kalimantan	Kalimantan	Rural	6%	61%	239
Kab. Ciamis	47.4%	West Java	Java	Rural	7%	51%	1168
Kab. Kutai Barat	46.2%	East Kalimantan	Kalimantan	Rural	9%	60%	146
Kab. Cianjur	45.8%	West Java	Java	Rural	10%	45%	2243
Kota Madiun	45.2%	East Java	Java	Urban	4%	80%	175
Kab. Kuningan	45.2%	West Java	Java	Rural	12%	67%	1055
Kota Banjarmasin	44.5%	South Kalimantan	Kalimantan	Urban	4%	55%	675
Kab. Barito Kuala	43.9%	South Kalimantan	Kalimantan	Rural	5%	62%	298
Melawi	43.9%	West Kalimantan	Kalimantan	Rural	13%	41%	196
**AVERAGE**					**8%**	**59%**	**645**
(e) Diabetes							
Kota Madiun	5.3%	East Java	Java	Urban	4%	80%	175
Kota Mojokerto	5.0%	East Java	Java	Urban	6%	80%	126
Kota Yogyakarta	4.8%	Jogyakarta	Java	Urban	7%	73%	412
Kab. Sidoarjo	4.6%	East Java	Java	Rural	6%	70%	2114
Kab. Gresik	4.5%	East Java	Java	Rural	12%	79%	1255
Kota Probolinggo	4.5%	East Java	Java	Urban	7%	72%	229
Kota Manado	4.5%	North Sulawesi	Sulawesi	Urban	5%	66%	425
Kota Surabaya	4.4%	East Java	Java	Urban	5%	66%	2847
Kep Seribu	4.4%	Jakarta	Java	Rural	12%	71%	23
Kota Jakarta Pusat	4.1%	Jakarta	Java	Urban	4%	55%	914
**AVERAGE**					**7%**	**71%**	**852**

*CVD* Cardiovascular Disease, *Urban* City, *Rural* District/Regent, *Pop* Population. The districts are ordered by risk factor prevalence

Table 5 further examines the disparities by three geographic and socioeconomic indicators, including region, income, and education. The absolute (relative) values show the difference (ratio) between Java and Papua as well as between the fifth and first quintiles. In all districts, the prevalence of obesity, hypertension, and diabetes are significantly higher among districts in Java, most affluent districts, and most educated districts. However, the prevalence of physical inactivity and smoking is significantly higher among districts in Papua and least educated districts. These results are similar among urban and rural districts, but those among urban are mostly not statistically significant.

**Table 5:** Geographic and socioeconomic disparity in CVD risk factors

	All districts (*n* = 514)					Urban (*n* = 97)					Rural (*n* = 417)				
	Smoking	Inactivity	Obesity	Hypertension	Diabetes	Smoking	Inactivity	Obesity	Hypertension	Diabetes	Smoking	Inactivity	Obesity	Hypertension	Diabetes
	[1]	[2]	[3]	[4]	[5]	[6]	[7]	[8]	[9]	[10]	[11]	[12]	[13]	[14]	[15]
Region															
Papua	27.8%	34.5%	27.3%	23.2%	1.1%	26.8%	40.7%	35.5%	24.9%	2.2%	27.9%	33.8%	26.4%	23.0%	1.0%
Sulawesi	27.9%	33.4%	32.8%	29.9%	1.9%	26.5%	36.5%	38.8%	28.8%	2.7%	28.1%	33.0%	31.8%	30.1%	1.7%
Kalimantan	27.3%	34.6%	28.1%	36.5%	1.9%	23.5%	43.6%	35.8%	34.7%	3.1%	28.0%	32.9%	26.6%	36.9%	1.6%
Sumatera	29.0%	33.8%	29.8%	27.2%	1.7%	27.3%	41.3%	35.3%	26.4%	2.4%	29.5%	31.7%	28.3%	27.4%	1.5%
Java	28.5%	31.3%	31.4%	34.9%	2.3%	27.5%	39.0%	37.9%	33.8%	3.2%	28.9%	28.4%	28.9%	35.3%	2.0%
Absolute	0.7%	−3.2%*	4.1%*	11.7%*	1.2%*	0.7%	−1.7%	2.4%	8.9%*	0.9%*	1.0%	−5.4%*	2.5%*	12.3%*	1.0%*
Relative	1.03	0.91	1.15	1.51	2.08	1.03	0.96	1.07	1.36	1.41	1.04	0.84	1.09	1.54	2.02
Income															
Q1 poor	27.9%	32.5%	26.9%	24.6%	1.2%	23.7%	34.8%	32.1%	26.9%	2.1%	28.0%	32.4%	26.7%	24.6%	1.2%
Q2	28.6%	31.5%	27.6%	29.2%	1.5%	28.4%	32.5%	33.1%	28.0%	2.5%	28.6%	31.5%	27.4%	29.3%	1.5%
Q3	29.2%	31.2%	29.9%	32.4%	1.9%	26.0%	42.1%	34.9%	28.5%	2.5%	29.7%	29.6%	29.2%	33.0%	1.8%
Q4	28.5%	33.9%	31.1%	30.8%	1.9%	26.7%	39.3%	35.9%	29.4%	2.8%	29.0%	32.5%	29.8%	31.2%	1.7%
Q5 rich	27.2%	37.5%	34.3%	31.9%	2.3%	27.2%	40.9%	38.1%	30.9%	2.8%	27.3%	33.7%	30.1%	32.9%	1.7%
Absolute	−0.7%	4.9%*	7.4%*	7.2%*	1.1%*	3.4%	6.1%	5.9%*	4.0%	0.7%	−0.7%	1.2%	3.4%*	8.3%*	0.6%*
Relative	0.98	1.15	1.27	1.29	1.92	1.14	1.17	1.18	1.15	1.33	0.97	1.04	1.13	1.34	1.47
Education															
Q1 least	29.1%	32.1%	25.4%	29.6%	1.1%	n/a	n/a	n/a	n/a	n/a	29.1%	32.1%	25.4%	29.6%	1.1%
Q2	28.4%	32.9%	28.9%	30.1%	1.7%	25.7%	38.0%	36.7%	31.4%	2.9%	28.7%	32.3%	28.0%	30.0%	1.5%
Q3	28.7%	33.6%	31.0%	30.1%	1.8%	26.2%	42.6%	36.5%	30.6%	2.8%	29.1%	31.8%	29.9%	30.0%	1.6%
Q4	27.8%	35.8%	31.7%	29.3%	2.0%	27.3%	40.3%	37.0%	29.4%	2.6%	28.0%	34.1%	29.7%	29.3%	1.8%
Q5 most	27.5%	32.1%	32.9%	29.9%	2.3%	27.1%	39.4%	36.5%	29.7%	2.8%	27.7%	27.5%	30.5%	30.1%	2.0%
Absolute	−1.6%*	0.0%	7.4%*	0.3%	1.2%*	n/a	n/a	n/a	n/a	n/a	−1.4%	−4.7%*	5.1%*	0.5%	0.9%*
Relative	0.94	1.00	1.29	1.01	2.05	n/a	n/a	n/a	n/a	n/a	0.95	0.85	1.20	1.02	1.77

*Q* Quintile, *CVD* Cardiovascular Diseases. Papua region includes Maluku and Nusa Tenggara. Income quintile used district-level poverty rate (e.g. Q1 = 20% of districts with highest poverty rate). Absolute (Relative) = Difference (Ratio) between Java and Papua as well as Q5 and Q1. Bold numbers with asterisk (*) show statistically significance at 5% level – full regression results are provided in Additional file 1 panels b-d

## Discussion

In Indonesia, we found a very high prevalence of CVD risk factors among men and women aged 10 years and above. For example, the prevalence of smoking, physical activity, obesity, hypertension ranged from 28 to 33% in 2018. We also found a huge geographic disparity in CVD risk factors. For instance, provinces and districts with the highest smoking prevalence are in the northern and southern parts of Sumatera, the western part of Kalimantan, the eastern and western parts of Java, the northern part of Sulawesi, and the southern part of Papua. We also found evidence of substantial socioeconomic disparity in CVD risk factors. The prevalence of obesity, hypertension, and diabetes is higher among urban and the richest and most educated districts, while that of physical inactivity and smoking is higher among rural and the least educated districts.

This disparity is similar to the global trend where the prevalence of obesity, hypertension, and diabetes is the largest in higher-income countries while that of smoking is largest in lower-income countries. However, our results show that obesity, hypertension, and diabetes are no longer exclusive to wealthier countries. In Indonesia, a lower-middle-income country, 40% of 514 districts have the prevalence of hypertension and obesity, ranging from 32 to 49% and 33 to 50%, respectively. This evidence highlights the need for generating similar findings in LMICs for policy evidence and national planning. Our results show that while the governments in LMICs are to continue tackling maternal mortality and infectious diseases (e.g. tuberculosis and dengue), they need to put more effort into the CVD prevention and control, especially in urban and the most developed and educated cities and regents.

Moreover, our findings show that all the bottom 10 districts with the lowest prevalence of diagnosed diabetes have zero prevalence, and nine of the districts are in the most eastern and least developed Papua region. On the other hand, all the top 10 districts with the highest prevalence of diabetes have a prevalence of 4.1% and above, and nine of the districts are in the most developed Java region. In addition to the issue with a considerable disparity between the two, the zero prevalence may also indicate the lack of health system ability to diagnosed diabetes in the least developed. The future national health survey cycle should also examine the undiagnosed diabetes to help confirm this.

There are at least two recommendations to reduce the CVD risk factors in Indonesia. First, there is a need for more comprehensive tobacco control efforts (e.g. ban of outdoor tobacco advertisements and plain packaging) to help reduce smoking, particularly in rural, more deprived and less educated districts. Secondly, comprehensive policies to promote a healthy diet is needed to help reduce obesity, hypertension, and diabetes in urban, wealthier, and most educated districts. Strategies may include taxes on unhealthy foods, subsidies on healthy foods, and regulations on salt, sugar and trans-fat [4].

Our study has two limitations. First, data on CVD risk factors by sex was not available for our analysis, which limited the disparity analysis among men and women. Second, data on the prevalence of hypertension were not broken up into diagnosed and undiagnosed, and that of diabetes was only for diagnosed. All this has limited our analysis of the disparity that is due to the lack of health system efforts in diagnosing CVD risk factors. Notwithstanding these limitations, our findings have important policy implications for Indonesia and beyond.

## Conclusion

The burden of CVD risk factors is high and increasing particularly among urban areas and districts with higher income and education levels. While the government needs to continue tackling the persistent burden from maternal mortality and infectious diseases, they need to put more effort into the prevention and control of CVDs and their risk factors.

## Supplementary information

**Additional file 1.**

## Data Availability

Available from the authors upon reasonable request.

## Abbreviations

Bappenas: National Planning Agency
BMI: Body Mass Index
CB: Census Block
CVD: Cardiovascular disease
DALY: Disability Adjusted Life Years
GPAQ: Global Physical Activity Questionnaire
LMICs: Low and middle-income countries
MET: Metabolic Equivalent of Task
OLS: Ordinary Least Square
Riskesdas: Basic Health Research
