# Supplementary material for: Geographic and socioeconomic disparity in cardiovascular risk factors in Indonesia: analysis of the Basic Health Research 2018
Source: BMC Public Health. 2020 Jun 26;20:1004. doi: 10.1186/s12889-020-09099-1 (PMC7318418; doi:10.1186/s12889-020-09099-1)
Supplement: Supplementary file 1 — Additional file 1. [file 12889_2020_9099_MOESM1_ESM.docx]

**SUPPLEMENTARY ONLINE APPENDIX**

The authors have provided this appendix to give readers additional information about their work.

Supplement to: Geographic and socioeconomic disparity of cardiovascular risk factors in Indonesia: Analysis of the Basic Health Research (Riskesdas) 2018

(Updated Apr 22, 2020)

**Contents**

Appendix 1. Regressions outputs of disparity CVD risk factors in Indonesia

Appendix 2. Regressions outputs of disparity CVD risk factors (Controlling for ag

**Appendix 1. Regressions outputs of disparity CVD risk factors in Indonesia**

|  |  | District | Smoking | | Inactivity | | Obesity | | Hypertension | | Diabetes | |
| --- | --- | --- | --- | --- | --- | --- | --- | --- | --- | --- | --- | --- |
|  |  | total | Coef. | (SE) | Coef. | (SE) | Coef. | (SE) | Coef. | (SE) | Coef. | (SE) |
|  |  | [1] | [2] |  | [3] |  | [4] |  | [5] |  | [6] |  |
|  |  |  |  |  |  |  |  |  |  |  |  |  |
| (a) Urban | |  |  |  |  |  |  |  |  |  |  |  |
|  | Rural | 417 | (reference) |  |  |  |  |  |  |  |  |  |
|  | Urban | 97 | **-1.74**** | (0.51) | **8.32**** | (1.25) | **8.27**** | (0.70) | 0.19 | (0.78) | **1.19**** | (0.09) |
|  |  | Cons | 28.62** | (0.22) | 68.24** | (0.54) | 28.43** | (0.30) | 29.78** | (0.34) | 1.56** | (0.04) |
|  |  |  |  |  |  |  |  |  |  |  |  |  |
| (b) Region | |  |  |  |  |  |  |  |  |  |  |  |
|  | Papua | 95 | (reference) |  |  |  |  |  |  |  |  |  |
|  | Java | 128 | 0.73 | (0.61) | **3.16**** | (1.56) | **4.09**** | (0.92) | **11.74**** | (0.69) | **1.22**** | (0.12) |
|  | Sumatera | 154 | **1.21**** | (0.59) | 0.71 | (1.50) | **2.49**** | (0.88) | **3.99**** | (0.66) | **0.55**** | (0.11) |
|  | Kalimantan | 56 | -0.49 | (0.76) | -0.14 | (1.94) | 0.78 | (1.14) | **13.36**** | (0.86) | **0.73**** | (0.15) |
|  | Sulawesi | 81 | 0.15 | (0.68) | 1.03 | (1.74) | **5.48**** | (1.03) | **6.70**** | (0.77) | **0.73**** | (0.13) |
|  |  | Cons | 27.77** | (0.46) | 65.53** | (1.18) | 27.27** | (0.70) | 23.19** | (0.52) | 1.13** | (0.09) |
|  |  |  |  |  |  |  |  |  |  |  |  |  |
| (c) Income/poverty | |  |  |  |  |  |  |  |  |  |  |  |
|  | Q1 poor | 102 | (reference) |  |  |  |  |  |  |  |  |  |
|  | Q2 | 103 | 0.72 | (0.63) | 0.99 | (1.59) | 0.74 | (0.91) | **4.61**** | (0.88) | **0.35**** | (0.12) |
|  | Q3 | 103 | **1.37**** | (0.63) | 1.35 | (1.59) | **3.04**** | (0.91) | **7.80**** | (0.88) | **0.72**** | (0.12) |
|  | Q4 | 103 | 0.64 | (0.63) | -1.43 | (1.59) | **4.23**** | (0.91) | **6.20**** | (0.88) | **0.73**** | (0.12) |
|  | Q5 rich | 103 | -0.66 | (0.63) | **-4.94**** | (1.59) | **7.38**** | (0.91) | **7.22**** | (0.88) | **1.11**** | (0.12) |
|  |  | Cons | 27.87** | (0.45) | 67.48** | (1.12) | 26.91** | (0.65) | 24.64** | (0.62) | 1.20** | (0.09) |
|  |  |  |  |  |  |  |  |  |  |  |  |  |
| (d) Education | |  |  |  |  |  |  |  |  |  |  |  |
|  | Q1 least | 103 | (reference) |  |  |  |  |  |  |  |  |  |
|  | Q2 | 103 | -0.65 | (0.63) | -0.78 | (1.60) | **3.46**** | (0.91) | 0.54 | (0.96) | **0.53**** | (0.12) |
|  | Q3 | 103 | -0.41 | (0.63) | -1.49 | (1.60) | **5.58**** | (0.91) | 0.56 | (0.96) | **0.69**** | (0.12) |
|  | Q4 | 103 | **-1.24**** | (0.63) | **-3.72**** | (1.60) | **6.28**** | (0.91) | -0.29 | (0.96) | **0.88**** | (0.12) |
|  | Q5 most | 102 | **-1.61**** | (0.63) | -0.02 | (1.61) | **7.43**** | (0.91) | 0.31 | (0.96) | **1.18**** | (0.12) |
|  |  | Cons | 29.07** | (0.44) | 67.88** | (1.13) | 25.44** | (0.64) | 29.59** | (0.68) | 1.13** | (0.09) |
|  |  |  |  |  |  |  |  |  |  |  |  |  |
|  | N | 514 | 514 |  | 514 |  | 514 |  | 514 |  | 514 |  |

Note: SE=Standard error; Q=Quintile; Coef=Coefficient; Cons=Constant. Income quintile used district-level poverty rate (e.g. Q1=20% of districts with highest poverty rate). For each risk factor, bivariate OLS regression was conducted to show associations between geographic and socioeconomic disparity in CVD risk factors. Bold numbers with ** show statistical significance at 5% level.

**Appendix 2. Regressions outputs of disparity CVD risk factors (Controlling for age)**

|  |  |  | District | Smoking | | Inactivity | | Obesity | | Hypertension | | Diabetes | |
| --- | --- | --- | --- | --- | --- | --- | --- | --- | --- | --- | --- | --- | --- |
|  |  |  | total | Coef. | (SE) | Coef. | (SE) | Coef. | (SE) | Coef. | (SE) | Coef. | (SE) |
|  |  |  | [1] | [2] |  | [3] |  | [4] |  | [5] |  | [6] |  |
|  |  |  |  |  |  |  |  |  |  |  |  |  |  |
|  | (a) Urban | |  |  |  |  |  |  |  |  |  |  |  |
|  |  | Rural | 400 | (reference) |  |  |  |  |  |  |  |  |  |
|  |  | Urban | 97 | **-1.74**** | (0.50) | **8.23**** | (1.23) | **8.29**** | (0.71) | 0.46 | (0.71) | **1.22**** | (0.09) |
|  |  |  | Cons | 28.23** | (0.48) | 35.54** | (1.17) | 28.07** | (0.68) | 23.68** | (0.68) | 1.06** | (0.09) |
|  |  |  |  |  |  |  |  |  |  |  |  |  |  |
|  | (b) Region | |  |  |  |  |  |  |  |  |  |  |  |
|  |  | Papua | 91 | (reference) |  |  |  |  |  |  |  |  |  |
|  |  | Java | 127 | 0.34 | (0.81) | 0.24 | (2.05) | **6.95**** | (1.21) | **9.39**** | (0.89) | **1.40**** | (0.16) |
|  |  | Sumatera | 151 | 0.96 | (0.62) | 0.53 | (1.57) | **3.50**** | (0.93) | **3.33**** | (0.69) | **0.64**** | (0.12) |
|  |  | Kalimantan | 55 | -0.77 | (0.77) | 0.34 | (1.95) | 1.14 | (1.15) | **13.13**** | (0.85) | **0.75**** | (0.15) |
|  |  | Sulawesi | 73 | 0.08 | (0.73) | -0.35 | (1.83) | **7.00**** | (1.09) | **5.96**** | (0.80) | **0.83**** | (0.14) |
|  |  |  | Cons | 27.73** | (0.60) | 37.21** | (1.51) | 29.14** | (0.90) | 21.30** | (0.66) | 1.20** | (0.12) |
|  |  |  |  |  |  |  |  |  |  |  |  |  |  |
|  | (c) Income/poverty | |  |  |  |  |  |  |  |  |  |  |  |
|  |  | Q1 poor | 98 | (reference) |  |  |  |  |  |  |  |  |  |
|  |  | Q2 | 94 | 0.72 | (0.67) | -0.41 | (1.66) | 1.32 | (0.98) | **3.13**** | (0.89) | **0.26**** | (0.13) |
|  |  | Q3 | 102 | 1.2 | (0.68) | 0.34 | (1.68) | **3.68**** | (0.99) | **5.54**** | (0.90) | **0.56**** | (0.13) |
|  |  | Q4 | 100 | 0.51 | (0.66) | 2.6 | (1.63) | **4.89**** | (0.96) | **4.67**** | (0.87) | **0.62**** | (0.13) |
|  |  | Q5 rich | 103 | -0.74 | (0.65) | **5.85**** | (1.61) | **7.87**** | (0.95) | **5.98**** | (0.86) | **1.03**** | (0.13) |
|  |  |  | Cons | 27.79** | (0.55) | 35.79** | (1.36) | 27.25** | (0.80) | 21.27** | (0.73) | 0.97** | (0.11) |
|  |  |  |  |  |  |  |  |  |  |  |  |  |  |
|  | (d) Education | |  |  |  |  |  |  |  |  |  |  |  |
|  |  | Q1 least | 97 | (reference) |  |  |  |  |  |  |  |  |  |
|  |  | Q2 | 99 | -0.8 | (0.64) | 0.98 | (1.62) | **3.80**** | (0.94) | -0.18 | (0.90) | **0.48**** | (0.12) |
|  |  | Q3 | 101 | -0.55 | (0.64) | 1.5 | (1.60) | **5.94**** | (0.93) | 0.25 | (0.90) | **0.69**** | (0.12) |
|  |  | Q4 | 102 | **-1.42**** | (0.63) | **3.51**** | (1.60) | **6.61**** | (0.93) | -0.41 | (0.89) | **0.90**** | (0.12) |
|  |  | Q5 most | 98 | **-1.76**** | (0.64) | -0.2 | (1.61) | **8.08**** | (0.93) | 0.44 | (0.90) | **1.23**** | (0.12) |
|  |  |  | Cons | 28.76** | (0.61) | 36.19** | (1.53) | 25.00** | (0.89) | 23.75** | (0.85) | 0.66** | (0.11) |
|  |  |  |  |  |  |  |  |  |  |  |  |  |  |
|  |  | N | 497 | 497 |  | 497 |  | 497 |  | 497 |  | 497 |  |

Note: SE=Standard error; Q=Quintile; Coef=Coefficient; Cons=Constant. Income quintile used district-level poverty rate (e.g. Q1=20% of districts with highest poverty rate). For each risk factor, bivariate OLS regression was conducted to show associations between geographic and socioeconomic disparity in CVD risk factors, controlling for district-level proportion of population 65+ years old (in quintile). Bold numbers with ** show statistical significance at 5% level.
